# Health enhancing behaviors in early adolescence: an investigation of nutrition, sleep, physical activity, mindfulness and social connectedness and their association with psychological distress and wellbeing

**DOI:** 10.3389/fpsyt.2024.1413268

**Published:** 2024-09-25

**Authors:** Kassie Bromley, Dashiell D. Sacks, Amanda Boyes, Christina Driver, Daniel F. Hermens

**Affiliations:** ^1^ Thompson Institute, University of the Sunshine Coast, Birtinya, QLD, Australia; ^2^ Department of Psychiatry and Behavioral Sciences, Boston Children’s Hospital, Boston, MA, United States; ^3^ Department of Psychiatry, Harvard Medical School, Boston, MA, United States

**Keywords:** sleep, adolescence, psychological distress, wellbeing, nutrition, physical activity, mindfulness, social connectedness

## Abstract

**Introduction:**

Nutrition, sleep and physical activity are termed the “big three” health enhancing behaviors (HEB) associated with psychological distress and wellbeing. This study sought to understand differential associations between an expanded group of HEB (nutrition, sleep, physical activity, mindfulness, social connectedness) and psychological distress/wellbeing in early adolescents.

**Methods:**

Correlational and regression analyses were conducted in N=103 (51% females) adolescents (12.6 ± 0.3 years of age) recruited from the Longitudinal Adolescent Brain Study.

**Results:**

Higher scores on sleep, social connectedness and mindfulness scales were significantly associated with lower psychological distress scores. While higher scores on social connectedness and mindfulness scales were significantly associated with higher wellbeing scores. When adjusting for sex, nutrition, sleep, social connectedness and mindfulness accounted for a significant proportion of variance in the psychological distress model whereas physical activity and social connectedness accounted for a significant proportion of the variance in the wellbeing model.

**Discussions:**

Overall findings make a strong case for expansion of the “big three” HEB to include mindfulness and social connectedness, especially given social connectedness emerged as the strongest predictor of both psychological distress and wellbeing. In addition, this research suggests that early adolescent nutrition, sleep quality, and mindfulness should be prioritized in efforts to reduce risk of difficulties, and physical activity prioritized as a protective factor for wellbeing in this population. Findings have implications for interventions, emphasizing the importance of addressing HEB factors comprehensively and tailoring strategies to the unique needs of early adolescents to foster positive mental health outcomes.

## Introduction

The adolescent years are a crucial period of transition from childhood to adulthood characterized by significant biological, psychological, and social changes. This developmental phase coincides with the peak onset of mental health difficulties. One third of adult mental health disorders begin by the age of 14, and more than half (62.5%) begin by 24 (1). Adolescent mental health aetiology has been widely explored over the past decade, with findings that reveal links between lifestyle factors, mental health and wellbeing (2–4). Lifestyle factors are often categorized as health enhancing behaviors (HEB) or health compromising behaviors (HCB), indicating their effects on mental health and wellbeing (5). Understanding the choices adolescents make regarding lifestyle factors, in the context of the rapid biological, psychological and social-environmental development is imperative, as they appear to be intimately connected and implicated in the aetiology of psychopathology. Furthermore, early and targeted interventions are key to preventing the progression of emerging mental health problems that may manifest into discrete mental health disorders (6). Early and benign interventions, that are effective, are particularly important in this pursuit, and thus HEBs offer an opportunity to positively impact mental health and wellbeing in adolescence.

The dynamic interplay between biological and psychological development occurring during adolescence underlies a drive for independence, greater freedom, and decision-making autonomy. Brain development typically involves amelioration of the so-called ‘maturational gap’ between cortical areas responsible for self-regulation and the subcortical areas responsible for emotional processing, which during adolescence heightens decision making’s sensitivity to emotional influences (7). At the same time, developing neural circuits responsible for decision-making are dynamically shaped by social experiences, such as peer interactions and social feedback (8). In addition, the brain’s reward system, which is involved in motivation and pleasure, undergoes significant changes, and appears to drive an increase in the salience of rewards leading to impulsive decision-making and risk-taking behavior (9, 10). Moreover, hormonal fluctuations during adolescence (particularly around pubertal onset) can impact mood, energy levels, and sleep patterns. These biological changes can influence lifestyle choices such as sleep habits, dietary preferences, and engagement in physical activity (11). Research indicates that adolescents may choose lifestyles that align with their developing identities, their peer acceptance, and their exploration of new experiences outside of parental influence (10). Of note, it has been demonstrated that while adolescents understand that their lifestyle choices can impact mental health and wellbeing, they are not motivated by promises of future improvements in their mental health, and instead their decisions are heavily influenced by contemporary social and emotional factors (10). Not surprisingly, during adolescence, HEB may not be prioritized (12–15). Hence, the patterns of engagement in HEB that emerge during adolescence are particularly important because they often lead to the establishment of enduring lifelong habits (12). In terms of mental health, there appears to be a bi-directional relationship between lifestyle factors and mental health. That is, engaging in HEB impacts mental health, and conversely mental health can influence engagement in HEB (16, 17). In addition, recent research indicates that lifestyle factors tend to combine, presenting cumulative protection (or risk) for mental health (18).

HEB are considered modifiable through behavior change, with relatively small changes in lifestyle behaviors resulting in improvements in mental health (19–23). Thus, they present critical avenues for early intervention and prevention. Understanding the contribution of HEB to mental health during adolescence is foundational to prevention and intervention efforts. Identifying which HEBs to target for the promotion of mental health, at key inflection points across the adolescent continuum has been a research direction advocated by several authors (12, 18, 20). While there has been a proliferation of research regarding the relationship between HEB and mental health and wellbeing over the past decade, many of these studies have focused on a singular HEB factor, failing to augment our understanding of the interconnected relationships between HEB. More recently, research has begun to quantify associations between multiple HEBs and mental health outcomes (4, 17, 20, 24–27). Three HEBs predominate this research and have recently been termed the “big three health enhancing behaviors” in relation their effect on mental health and wellbeing in a young adult population (28).

In a study investigating the differential and higher-order associations between ‘the big three’ health behaviors (sleep, physical activity, and dietary factors) as predictors of psychological distress and well-being in young adults (18-25yrs), sleep quality was found to be the most important predictor of psychological distress and well-being whereas physical activity and diet were found to be secondary but still significant factors (28). The authors suggested that interventions targeting sleep, diet, and physical activity within young adult populations may help to promote optimal mental health and well-being within this at-risk population, as targeting one individual behavior may not always improve other health behaviors. The roles of multiple HEBs, in terms of their combined impact on mental health measures, has also been examined in youth. In their systematic review of 28 studies in young people aged 11-19 years, Hale et al. (29) found that multicomponent interventions, typically involving physical activity with other features (e.g. nutrition), were useful in improving psychological well-being and quality of life, with mixed evidence for self-esteem, and limited evidence in terms of the impacts on psychological ‘ill-being’ (as there were few studies that addressed this). In a more recent systematic review, Wilhite et al. (30) found that across 141 studies, children (5-13 years) and adolescents (14-17 years) with the most favorable mental health outcomes were more active, less sedentary, and had longer sleep duration. Similarly, a study of 315 students (mean age 11.4 years) from two British schools in Dubai, found distinct profiles, based on combinations of physical activity, screen time, sleep quality, and dietary habits, were related to mental health and school satisfaction outcomes; for example, those with poor sleep and poor diets had the worst outcomes (31). Consistent again, a study involving over 30,000 Chinese children and adolescents (mean age 12.4 years), found that those who reported having multiple ‘unhealthy lifestyle behaviors’ (i.e., poor diet, lack of sleep, decreased outdoor activity and increased screentime) were more likely to have increased emotional and behavioral problems (32).

The above-mentioned studies in both young adults and in children/adolescents demonstrate that combinations of HEBs (typically the ‘big three’, but not necessarily) are associated with mental health measures. Despite mostly promising evidence, pointing to multicomponent intervention strategies, it remains unclear what combination of HEBs lead to better outcomes and whether the addition of other potential HEBs to the ‘big three’ can enhance mental health and wellbeing. That is, in addition to sleep, physical activity and nutrition, it may be important to understand how a larger combination of lifestyle factors relate to mental health and wellbeing, particularly within the adolescent population, where onset rates of mental health disorders are at their highest. As noted above, adolescent decision making (important in the adoption of behaviors) is heavily influenced by contemporary social (externalizing) and emotional (internalizing) factors. Hence, we suggest that two additional HEBs to evaluate in youth are social connectedness and mindfulness, both outlined below.

Social connectedness is important during adolescence, a period characterized by a heightened need for peer acceptance and social belonging (33, 34). Individuals with high levels of social connectedness appear better equipped to cope with stress and exhibit higher levels of wellbeing (35–37). Moreover, social connectedness is increasingly acknowledged for its significant impact on mental health, with research consistently showing associations between social isolation and psychological distress (34, 35, 38, 39). Of note, there is evidence that higher social connectedness in early adolescence may be protective for mental health for those experiencing cyberbullying and/or cybervictimization (40). However, despite being key aspect of adolescent development, social connectedness is often underrepresented in HEB frameworks. Mindfulness, acknowledged as both a modifiable trait and practice, has demonstrated promising outcomes in enhancing emotional regulation and protecting against stress (41–45). This practice encourages present-moment awareness and emotional regulation, which are crucial during the rapid developmental changes of adolescence (46). While mindfulness practices have been increasingly recognized for their benefits in reducing anxiety, depression, and stress among adolescents its status as a HEB in early adolescents remains an area requiring further investigation (41, 45).

This study aims to expand on the important research generated to date by investigating the relationships between a novel combination of five HEBs - nutrition, sleep, physical activity, mindfulness, and social connectedness – in terms of their association with psychological distress & wellbeing measures in early adolescents. Mindfulness and social connectedness have been less frequently examined and have yet to be examined in combination with the “big three” HEB in this age group. As outlined above, the inclusion of social connectedness and mindfulness is based on emerging evidence suggesting that these factors also play a critical role in mental health and wellbeing during adolescence. When considering these HEBs to include in this novel expansion, recent research and theoretical models that support the integration of these constructs into a comprehensive HEB framework was considered. It is particularly relevant to expand the combination of HEBs that may relate to mental health in early adolescents along these lines, as this age group is increasingly navigating complex social and emotional landscapes (8, 44). Thus, in this study, we hypothesized that higher scores among all five HEB factors would be significantly associated with lower levels of psychological distress and higher levels of wellbeing, accordingly to bivariate analyses. We further hypothesized that, in combination, HEB factors would be significant predictors of psychological distress and wellbeing via regression modelling.

## Methods

### Study design and setting

The Longitudinal Adolescent Brain Study (LABS) is a prospective cohort study of high school students from the Sunshine Coast region in Australia (47, 48). LABS commenced in July 2018 and has 3 key aims; (1) to identify early manifestations of mental ill health in adolescents; (2) to longitudinally monitor changes in the adolescent brain in conjunction with social and lifestyle influences impacting mental wellbeing; and (3) to identify opportunities for early intervention in young people experiencing signs and symptoms of mental health problems. Participants are assessed at baseline (timepoint 1) and invited to return for assessments every four months, across five years, until the last year of high school. At each time-point the participants complete a self-report questionnaire, cognitive testing, neuropsychiatric interview, and neuroimaging (electroencephalography and magnetic resonance imaging). This study reports on the self-report questionnaires, collected at baseline, to determine cross-sectional relationship between measures in participants at the time of study commencement. Ethical approval was granted by the University of the Sunshine Coast (UniSC). To satisfy requirements listed in the National Statement, assent is always obtained from both the prospective participants and consent from their caregiver, with assent of the young person always taking priority over their caregiver. In alignment with the National Statement, caregivers who provided standing consent were notified of each assessment session (via preferred method) and reminded that they may withdraw consent for the project, or their standing consent, at any time.

### Participants

All data was collected onsite at the Thompson Institute, University of the Sunshine Coast, Australia. Participants who were 12-13 years of age, in grade 7 (first year of high school) and proficient in spoken and written English, met inclusion criteria. Participants who reported suffering from a major neurological disorder (such as epilepsy), intellectual disability, major medical illness or who reported having sustained a head injury (with loss of consciousness for exceeding 30 minutes) were excluded. Recruitment was conducted via a range of channels, including (but not limited to) local press (print media, television, and radio), community services and events (youth groups, churches, headspace, etc.), schools (e.g. newsletters and research team engagement/presentations), UniSC/TI websites and newsletters (for example, LABS website and LABS/other projects updates/newsletters), and social media (e.g., Facebook).

### Measures

The self‐report questionnaire was administered via touch-screen tablet computer. This study used a subtest of scales selected from the larger LABS self-report survey.

#### Psychological distress

Psychological distress was measured using the Kessler Psychological Distress Scale (K10), a 10-item self-report questionnaire asking about feelings over the past 30 days, which measures depression and anxiety levels on a five-point Likert scale. Individual scores are tallied providing an overall score ranging from 10-50, with higher scores indicating increased psychological distress. The Kessler Psychological Distress Scale has been validated in 2967 children and adolescents aged 11-17 years and has been shown to have good reliability and validity across cultures and clinical populations (49–51).

#### Wellbeing

Wellbeing was measured using the Composure Own-worth Mastery Positivity Achievement Satisfaction – Wellbeing (COMPAS-W) scale, which comprises 26 items, scored on a five-point Likert scale, and assesses poor to optimal subjective and psychological wellbeing (52). The total score ranges from 26-130, with higher scores indicating greater wellbeing. The Composure Own-worth Mastery Positivity Achievement Satisfaction – Wellbeing scale has been validated for use in Australian adults (18-61 years) and Australian youth aged 10-17years, with high internal consistency (52, 53).

#### Sleep

Participants completed the Pittsburgh Sleep Quality Index (PSQI), a 19-item retrospective (past month) questionnaire. The Pittsburgh Sleep Quality Index measures seven subscales of sleep: sleep quality, sleep latency, sleep duration, habitual sleep efficiency, sleep disturbance, use of sleep medication, and daytime dysfunction. Responses on a four-point Likert scale are tallied to give an overall score, ranging from 0-21. Lower scores indicate better sleep quality, and higher scores indicate sleep dysfunction. The Pittsburgh Sleep Quality Index has been used in numerous previous studies involving child and adolescent samples and has shown to be a reliable and valid measure of sleep quality and quantity (54, 55). Items seven and ten were altered as they are not universally applicable for adolescent populations. Item seven, assessing trouble with ‘staying awake while driving, eating meals, or engaging in social activity’, was altered by replacing ‘driving’ with ‘studying’. Item ten was omitted as it relates to bed partners or roommates.

#### Eating habits

Participants completed six items from the Food Frequency Questionnaire (FFQ), according to the protocol utilized in the Healthy Behavior School Aged Children Study (HBSC) (56). Scores range from 0-31, with higher scores indicating healthier eating habits. The FFQ was selected for its ability to succinctly assess weekly eating habits while being appropriate for cross-cultural use in comparison to alternate scales (e.g., the YRBS which was developed for American use). The Food Frequency Questionnaire has been used and validated with 11-12‐year old adolescents in Belgium and Italy (57).

#### Physical activity

Participants completed the Moderate to Vigorous Physical Activity Scale (MVPA), which consists of three items to measure physical activity in the past seven days. It contains one Moderate Physical Activity item (MPA) and two Vigorous Physical Activity items (VPA). Scores range from 0-18 with higher scores indicating healthier levels of physical activity (58). The Moderate Physical Activity item was validated in 15‐17-year-old Australian adolescents and the Vigorous Physical Activity items were validated in 13- and 15-year-old Australian students (59, 60).

#### Mindfulness

Participants completed the 14-item Mindful Attention Awareness Scale – Adolescents (MAAS-A) scale which uses a six-point Likert scale to measure awareness of, and attention to what is taking place in the present. Scores range from 14-84 with higher scores indicating higher (healthier) mindfulness traits. The Mindful Attention Awareness Scale – Adolescents was developed with 595 adolescents aged 14–18 years from normative and psychiatric outpatient samples (61). The measure has been validated in a Dutch sample of 717 adolescents aged 11–17 years (62).

#### Social connectedness

Participants completed the Social Connectedness Scale (SCS) which consists of 15 items on a six-point Likert scale. Scores range from 15-90 with higher scores indicating that participants felt more socially connected. The Social Connectedness Scale was validated in a population of college students and has been administered to adolescents in an online context (3, 12, 63).

### Statistical analysis

SPSS^®^ Version 26 (SPSS Inc., Chicago, Illinois, USA) was used for statistical analysis. Spearman’s rho correlations were used for the correlational analyses as the scores for variables were not normally distributed. Given that four of the five HEB variables were scored in one direction, that is, higher scores equated to better HEBs, the PSQI scores were transformed (multiplied by -1) for correlational and multiple regression analysis, to be consistent with the other four HEB. Two separate standard multiple regression analyses were performed, one with K10 and the other with COMPAS-W entered as the dependent variable. In both models, MVPA, FFQ, PSQI, SCS and MAAS‐A scores were entered as the predictor variables. Regression assumptions were deemed as met following visual inspection of the normal P-P plot and scatterplot of the residuals and given the VIF statistics were all under 3. Significance level for regression analyses was set at *p*<.05, however, due to multiple comparisons the significance threshold for the correlation analyses was *p*<.005 (Bonferroni corrected; 0.05/9).

## Results

**Table 1** provides the sample descriptive statistics.

**Table 1:** Descriptive statistics for the sample of early adolescents N=103.

	Min	Max	Mean	Possible Response Range	*SD*
**N=103 (51% Female: 49% Male) Age (Years)**	12.04	13.13	12.62	12-13	0.29
**Psychological Distress (K10)**	10	35	16.04	10-50	5.51
**Wellbeing (COMPAS-W)**	77	120	100.63	26-130	11.51
**Sleep (PSQI)**	0	15	4.96	0-21	2.76
**Nutrition (FFQ)**	12	30	22.96	0-31	3.49
**Physical Activity (MVPA)**	1	18	12.08	0-18	3.58
**Mindfulness (MAAS-A)**	16	84	69.26	14-84	11.16
**Social Connectedness (SCS)**	24	90	73.53	15-90	13.20

PSQI scores are not transformed (multiplied by -1) in **Table 1**.

PSQI, Pittsburgh Sleep Quality Index; FFQ, Food Frequency Questionnaire; MVPA, Moderate-to-Vigorous Physical Activity scale; MAAS-A, Mindfulness Attention Awareness Scale – Adolescents; SCS, Social Connectedness Scale; K10, Kessler Psychological Distress scale; COMPAS-W, Composure Own-worth Mastery Positivity Achievement Satisfaction – Wellbeing scale.

### Correlational analysis

Correlation results are presented in **Table 2**. Spearman’s rho correlations indicated significant negative relationships (*p*<.001) between K10 and three HEB variables of sleep, social connectedness and mindfulness (PSQI [transformed], SCSS and MAAS-A). There was no association between K10 and HEB of physical activity and nutrition (MVPA and FFQ). A significant (*p*<.001) positive relationship was observed between COMPAS-W HEB variables of social connectedness and mindfulness (MAAS-A and SCS). There was no association between COMPAS-W and HEB of physical activity, nutrition and sleep (MVPA, FFQ and PSQI).

**Table 2:** Correlation coefficients between K10, COMPAS- W and individual HEB N=103.

	Physical Activity (MVPA)	Nutrition (FFQ)	Sleep (PSQI)	Social Connectedness (SCS)	Mindfulness (MAAS-A)	Wellbeing (COMPAS-W)
**Psychological Distress (K10)**	-.181	-.228	-.548^***^	-.584^***^	-.616^***^	-.511
**Wellbeing** **(COMPAS-W)**	.318	.236	.313	.574^***^	.411^***^	

^***^Significant at *p*<.001. PSQI scores were transformed (multiplied by -1) to be consistent with the other four HEB variables.

Sleep, Pittsburgh Sleep Quality Index (transformed); Nutrition, Food Frequency Questionnaire; Physical activity, Moderate-to-Vigorous Physical Activity scale; Mindfulness, Mindfulness Attention Awareness Scale - Adolescents; Social connectedness, Social Connectedness Scale; Psychological distress, Kessler Psychological Distress scale.

### K10 model

The Model Summary R square statistics showed that the regression model explained 50% of the variance in K10 scores, and the ANOVA output of the multiple regression analysis showed that this proportion of the variance was significant *F*(5,94) = 18.46, *p<*.001. **Table 3** shows the contribution of each independent variable to the overall regression model. The multiple regression revealed that the transformed PSQI (*p=*<.001) and the SCS (*p=*<.001) each contributed significantly to the regression model, while the MVPA (*p=*.344), the FFQ (*p=*.103) and the MAAS-A (*p=*.100) did not make significant contributions.

Table 3ARegression coefficeints for predicting psychological distress (K10) with 5 predictors N=103.

**Table:** 

	B	Std err	β	*t*	sig.	sr^2^	Tolerance	VIF
**Physical Activity (MVPA)**	.115	.121	0.075	.951	.344	.005	.865	1.156
**Nutrition (FFQ)**	-.203	.123	-0.129	-1.648	.103	.015	.883	1.133
**Sleep (PSQI)**	-.656	.169	-0.329	3.875	<.001^***^	.081	.743	1.345
**Social Connectedness (SCS)**	-.144	.041	-0.345	-3.477	<.001^***^	.065	.547	1.829
**Mindfulness (MAAS-A)**	-.082	0.49	-0.166	-1.661	.100	.015	.540	1.851

**Table 3B:** Regression coefficeints for predicting psychological distress K10 with 6 predictors N=103.

	B	Std err	β	*t*	sig.	sr^2^	Tolerance	VIF
**Physical Activity (MVPA)**	.176	.121	.115	1.456	.149	.011	.826	1.211
**Nutrition (FFQ)**	-.257	.122	-.163	-2.100	.038*	.022	.852	1.173
**Sleep (PSQI)**	-.547	.171	-.275	3.192	.002**	.052	.691	1.448
**Social Connectedness (SCS)**	-.134	.041	-.321	-3.307	.001**	.056	.541	1.848
**Mindfulness (MAAS-A)**	-.105	.049	-.212	-2.139	.035*	.023	.519	1.927
**Sex (f, m)**	-2.006	.844	-.183	-2.376	.020*	.029	.864	1.158

^*^Significant at *p*<.05; ^**^Significant at *p*<.01; ^***^Significant at *p*<.001.

K10, Kessler Psychological Distress scale; MVPA, Moderate-to-Vigorous Physical Activity scale; FFQ, Food Frequency Questionnaire; PSQI, Pittsburgh Sleep Quality Index (transformed); SCS, Social Connectedness Scale; MAAS-A, Mindfulness Attention Awareness Scale – Adolescents; sr^2^, semi-partial correlations squared; VIF, Variance Inflation Factor.

Follow-up regressions were conducted to determine whether the overall model would remain the same with sex (female/male) entered as a covariate. In predicting psychological distress, the summary R square statistics showed that the regression model explained 52.4% of the variance in K10 scores, and the ANOVA output of the multiple regression analysis showed that this proportion of the variance was significant F(6,93) = 17.08, *p*<.001. The multiple regression revealed that Sex (*p*=.020) along with the FFQ (*p*=.038), transformed PSQI (*p*=.002), SCS (*p*=<.001) and the MAAS-A (*p*=.035) contributed significantly to the K10 model, while the MVPA (*p*=.149) did not make a significant contribution.

### COMPAS-W model

The Model Summary R square statistics showed that the regression model explained 40% of the variance in COMPAS-W scores, and the ANOVA output of the multiple regression analysis showed that this proportion of the variance was significant *F*(5,94) = 12.75, *p<*.001. **Table 4** reports the contribution of each independent variable to the overall regression model. The multiple regression revealed that the MVPA (*p=*.032) and SCS (*p=*<.001) contributed significantly to the regression model, while the FFQ (*p=*.161), transformed PSQI (*p=*.701) and MAAS-A (*p=*.831) did not make significant contributions.

Table 4ARegression coefficeints for predicting wellbeing (COMPAS-W) with 5 predictors N=103.

**Table:** 

	B	Std err	β	*t*	sig.	sr^2^	Tolerance	VIF
**Physical Activity (MVPA)**	.599	.275	0.186	2.175	.032^*^	.030	.865	1.156
**Nutrition (FFQ)**	.395	.280	0.120	1.414	.161	.013	.883	1.133
**Sleep (PSQI)**	.148	.384	0.036	.386	.701	.001	.743	1.345
**Social Connectedness (SCS)**	.444	.094	0.509	4.726	<.001^***^	.141	.547	1.829
**Mindfulness (MAAS-A)**	.024	.132	0.023	.214	.831	.000	.540	1.851

**Table 4B:** Regression coefficeints for predicting wellbeing COMPAS-W with 6 predictors N=103.

	B	Std err	β	*t*	sig.	sr^2^	Tolerance	VIF
**Physical Activity (MVPA)**	.715	.277	.222	2.576	.012*	.041	.826	1.211
**Nutrition (FFQ)**	.293	.280	.089	1.044	.299	.007	.852	1.173
**Sleep (PSQI)**	.355	.393	.085	-.904	.368	.005	.691	1.448
**Social Connectedness (SCS)**	.462	.093	.530	4.972	<.001***	.152	.541	1.848
**Mindfulness (MAAS-A)**	-.020	.112	-.019	-.178	.859	.000	.519	1.927
**Sex (f, m)**	-3.815	1.934	-.166	-1.973	.051	.024	.864	1.158

^*^Significant at *p*<.05; ^***^Significant at *p*<.001.

COMPAS-W, Composure Own-worth Mastery Positivity Achievement Satisfaction – Wellbeing scale; MVPA, Moderate-to-Vigorous Physical Activity scale; FFQ, Food Frequency Questionnaire; PSQI, Pittsburgh Sleep Quality Index (transformed); SCS, Social Connectedness Scale; MAAS-A, Mindfulness Attention Awareness Scale – Adolescents; sr^2^, semi-partial correlations squared; VIF, Variance Inflation Factor.

Follow-up regressions were conducted to determine whether the overall model would remain the same with sex (female/male) entered as a covariate. In predicting wellbeing, the summary R square statistics showed that the regression model explained 42.8% of the variance in COMPAS-W scores, and the ANOVA output of the multiple regression analysis showed that this proportion of the variance was significant F(6,93)=11.59, *p*<.001. The multiple regression revealed that the MVPA (*p*=.012) and SCS (*p*=<.001) contributed significantly to the COMPAS-W model, while sex (*p*=.051) FFQ (*p*=.299) along with the transformed PSQI (*p*=.368), MAAS-A (*p*=.859) did not make a significant contribution.

## Discussion

This study is the first to explore the potential contributions of a unique combination of five HEB variables (Nutrition, Sleep, Physical Activity, Mindfulness and Social Connectedness) to the psychological distress and wellbeing of early adolescents. The findings here add valuable insights that may inform targeted prevention and intervention efforts for adolescent mental health and wellbeing. Three of the five HEBs, including sleep, social connectedness and mindfulness demonstrated significant negative associations with psychological distress. Two HEBs including social connectedness and mindfulness demonstrated significant positive associations with wellbeing. Furthermore, regression modelling revealed that sex may play a role in modifying the relationship between HEBs and psychological distress but not wellbeing. Specifically, both sleep and social connectedness were significant predictors of psychological distress before sex was added to the model. After adding sex, nutrition, sleep, social connectedness and mindfulness (along with sex) were significant predictors. Whereas physical activity and social connectedness remained the only two predictors of wellbeing despite sex being added as a 6^th^ predictor of the model. This suggests that unique combinations of these HEBs may relate to different mental health and wellbeing outcomes in early adolescence. In this regard, the findings here provide important evidence for the field and by expanding the number HEBs analyzed, this study suggests a key role for social connectedness among a combination of HEBs that may be targeted for early intervention approaches to mental health problems in this age group. The following paragraphs discuss the findings for the various HEBs, in the context of the extant literature.

The relationship between sleep and mental health has also been previously reported in adolescent populations (64). Earlier studies focused on sleep duration as a predictor of mental health, whereas more recent studies utilized measures of sleep quality, rather than sleep duration alone, arguing that relationships between sleep and mental health may be more complex than simply duration of sleep (65, 66). This study acknowledges nascent research and as such, the PSQI total score was utilized to measure several important sleep variables resulting in a total sleep score. In this study, participants who scored higher on the SCS scored lower on the K10, indicating that those who perceive themselves to be more socially connected, had decreased psychological distress. This finding confirms the association between social connectedness and psychological distress in the adolescent population (33, 34). Participants who scored higher on the MAAS-A tended to score lower on the K10, suggesting that ‘healthier’ mindfulness traits or behaviors may be associated with decreased psychological distress. There is a currently a relatively limited body of literature regarding the relationship between mindfulness and psychological distress specifically within the adolescent population (41, 45). Our finding highlight a need for further research regarding the mechanism of action and strategies for modifying trait mindfulness for improved mental health. Furthermore, future studies that utilize measures such as the MAAS-A should endeavor to parse out whether the reported levels of mindfulness are trait-like and/or amenable to various interventions that target mindfulness behaviors. Taken together, our findings from correlational analysis between the K10 and HEBs of sleep, social connectedness and mindfulness align with previous research and suggest that there may be some generalizability of these associations in the early adolescence phase (2, 4, 18, 39, 41, 45, 67–71)

While the finding that there was not a significant relationship between physical activity and psychological distress during early adolescence was contrary to our hypothesis, this finding is somewhat in accord with existing research demonstrating mixed evidence about this relationship, especially among longitudinal research studies (72–74) In a recent example of this, Ganjeh et al. (74) conducted a longitudinal study to assess whether physical activity prevents the development of mental health problems in children and adolescents aged 0-12 years. This study found that while higher levels of physical activity were associated with better general mental health at the next time point, these effects were not observed from preadolescence to adolescence. Indeed, several other studies have demonstrated that the relationship between physical activity and mental health is not linear across the lifespan (73). Ganjeh et al. (74) suggest that rapid biopsychosocial development is likely to mediate the effect that physical activity has on mental health during this time. Nigg et al. (75), present a range of other potential factors to explain mixed findings in the adolescent population. These include the notion that the effects of physical activity are context specific, such as being part of a group or team, and that physical activity may not always have a positive effect on the mental health of adolescents because of social/environmental factors such as teasing/bullying. Concurrently, the issue of reliability must be taken into account, as there is evidence suggesting that the accuracy of self-reported physical activity varies with age (76). In this regard, older participants tend to exhibit greater reliability in reporting their physical activity levels. Hence, the observed age-dependent outcome could potentially be attributed to reliability concerns in this study.

In relation to wellbeing, findings from this study reveal that HEB factors of social connectedness and mindfulness demonstrated a significant positive relationship with wellbeing. These findings contribute to the growing body of literature exploring the multifaceted nature of wellbeing and its links to HEB (25, 77–80). Mindfulness has been referenced as a pathway to adolescent emotional well-being (45, 81). Consistent with previous studies, adolescents who indicated higher trait mindfulness also reported higher levels of wellbeing. Participants who indicated feeling more socially connected had higher levels of wellbeing and this is an important finding because while the relationship between social connectedness and mental illness has been studied in adolescence, the relationship between social connectedness and wellbeing has been neglected in the literature (39). This study provides strong evidence for the link between social connectedness and wellbeing during early adolescence.

It is worth noting that several correlations among HEBs in this study approached significance (with correlation coefficients above/below ±0.3). That is, physical activity and sleep both demonstrated positive associations with well-being, albeit non-significant after Bonferroni correction. Future research with larger sample sizes should further investigate these associations. Past evidence along with current evidence from this study strongly supports the continued exploration of these relationships. This study has highlighted the need to explore other potential HEB in addition to the “big three” to understand the combined influence on mental health and wellbeing, particularly within the adolescent population, where onset rates of mental health disorders are at their highest. The idea that HEB should be targeted within preventative and intervention programs to promote optimal mental health and wellbeing in adolescence is not new. Nutrition, sleep and exercise most often feature in these programs, yet this study demonstrates that it is important to explore other potential HEB outside of the “big three”. The five HEBs explored in this study are likely to offer a range of protective benefits contributing to adolescent mental health and wellbeing. The regression modelling suggests, however, that the protective benefits of all five HEB may not be equal. Interestingly, in both models, social connectedness was found to be an important predictor, combining with nutrition, sleep and mindfulness in the psychological distress model and with physical activity in the wellbeing model to explain most of the variance. Hence, the combinatorial impact of these groups of HEBs may be a line of inquiry in future research (and interventions) (24).

Given the findings of the follow-up regression models, adjusting for sex as a covariate, there may be differential relationships between HEB and psychological distress for females versus males. Results suggest that while social connectedness and sleep are consistent predictors of psychological distress across genders, nutrition, sleep and mindfulness have differential impacts on psychological distress depending on female or male sex. However, no such differences were found for wellbeing, indicating that sex may not significantly influence the relationship between HEB and wellbeing outcomes in early adolescents. In terms of individual HEB, social connectedness emerged as the strongest predictor of both psychological distress and wellbeing regardless of sex. In the context of adolescent health promotion, this points to social connectedness as a priority target to address as a step zero or alongside traditional mental health and wellbeing prevention and intervention programs in the early adolescent population (38, 39, 82). In addition, this research suggests that early adolescent sleep quality should be prioritized in efforts to reduce risk of mental health difficulties, and physical activity prioritized as a protective factor for wellbeing in this population.

Of note, this study is unique in its consideration of both psychological distress and wellbeing as dependent variables, providing insights into how HEBs impact both positive and negative dimensions of mental health in early adolescents. Moreover, our study employs the Kessler Psychological Distress Scale (K10) scale to assess psychological distress, a broader measure of mental health which is distinct from other studies that have measured symptoms of specific mental health disorders such as anxiety and depression. This is particularly significant as psychological distress is considered a precursor to mental disorders, and a substantial proportion of youth experience high levels of psychological distress but may not meet criteria for a specific mental health diagnosis. For instance, according to the 2022 Mission Australia Youth Survey, 28.8% of individuals aged 15-19 reported high levels of psychological distress (83). Similar to those with a diagnosed mental disorder, young individuals experiencing subthreshold depression and anxiety are prone to lower quality of life, functional difficulties, heightened distress, greater likelihood of suicidal ideation and an elevated risk of poor mental health in later life (84). By capturing a broader measure of mental health, as well as a measure of wellbeing, our study supports a more nuanced exploration of the impact of HEB. Unlike many previous studies that primarily focus on adults or young adults, our research targets a critical developmental period—early adolescence (12-13 years old)—which presents unique challenges and opportunities the related to lifestyle habit formation and adolescent mental health. By examining this novel combination of five HEB factors, we aim to gain valuable insights into their individual and cumulative effects on adolescent mental health outcomes to inform targeted prevention and intervention efforts.

Wellbeing and psychological distress are considered to be positive and negative dimensions of mental health, respectively. While this study did not set out to directly explore the relationship between these constructs, the findings do generate discussion about how these constructs relate to each other and the combination of HEBs that may be associated with these constructs. We found that the psychological distress (K10) and wellbeing (COMPAS-W) measures were associated with each other in early adolescents, as evidenced by a strong significant negative correlation. However, the evidence that a different combination of HEB factors predict each of these mental health indexes, supports findings that they are not entirely overlapping constructs (85). Furthermore, the different combination of HEB associated with greater risk for psychological distress versus protection for wellbeing is important because it suggests that focusing solely on promoting wellbeing may not be sufficient to address mental health issues in adolescents. While enhancing wellbeing is undoubtedly valuable, understanding and addressing factors that may be distinctly related to psychological distress is also crucial. Thus, shifting our perspective on HEB is imperative, as it requires us to recognize that these HEBs are not just lifestyle enhancements but may be key drivers underlying protective/risk factors for mental health or ill health.

Some limitations of the current study need to be acknowledged. The use of self-report measures for all variables may be impacted by factors such as insight and honesty, social desirability bias and question fatigue. In addition, there is a possibility of self-selection bias in the sample due to recruitment by expression of interest. This study did not measure ethnicity or socioeconomic status within our sample, which may impact the generalizability of the findings. Finally, although reporting data from a single time-point provides crucial information about the relationship between these variables at the onset of adolescence, it may not represent the relationship of these variables throughout adolescence. By examining this baseline data, we can gain preliminary insights into the trends and patterns emerging within the LABS sample. The aim of this initial analysis is to help identify the cross-sectional relationships between HEB and mental health and wellbeing measures in participants at the time of study commencement. LABS is an ongoing research study, with a goal of recruiting 500 participants. To fully explore the associations presented here, we aim to further research the relationships between HEBs and mental health measures, with larger sample sizes, over multiple time-points. Planned future longitudinal analyses would enhance our understanding of the patterns of relationships between variables throughout the adolescent years. A better understanding of how HEBs contribute individually and collectively to psychological distress and wellbeing along the adolescent continuum could assist in shaping preventive programs and intervention strategies to enhance mental health and wellbeing during this critical phase of life. With replication of these findings and further development of this research, there is potential for future applications such as the utilization of these HEB metrics to determine an individual’s level of protection/risk for good/poor mental health and wellbeing and therefore help individuals make more informed decisions about their lifestyle factors.

In conclusion, this study provides valuable insights into the relationships between HEB factors, psychological distress, and wellbeing in 12-13-year-old adolescents. The findings support previous research and make a strong case for the expansion of the “big three” HEBs to include mindfulness and social connectedness, especially given social connectedness emerged as the strongest predictor of both psychological distress and wellbeing. These findings have implications for interventions, emphasizing the importance of addressing HEB factors comprehensively and tailoring strategies to the unique needs of early adolescents to foster positive mental health outcomes.

## Data Availability

The raw data supporting the conclusions of this article will be made available by the authors, without undue reservation.
